# Supplementary material for: Chromosome Microarray Analysis and Exome Sequencing: Implementation in Prenatal Diagnosis of Fetuses with Digestive System Malformations
Source: Genes (Basel). 2023 Sep 26;14(10):1872. doi: 10.3390/genes14101872 (PMC10606699; doi:10.3390/genes14101872)
Supplement: Supplementary file 1 [file genes-14-01872-s001.zip › Supplementary file S1.pdf]

Supplementary file S1. Relational database.

A.

The referenced databases included internal databases of sample laboratories and online international public databases, such as the Online Mendelian Inheritance in Man (OMIM, http://www.omim.org), DECIPHER (http://decipher.sanger.ac.uk/), database of genomic variants (DGV, http://dgv.tcag.ca/dgv/app/home), the International Standards for Cytogenomic Arrays (ISCA, https://www.iscaconsortium.org/), and the University of California, Santa Cruz genome browser (http://genome.ucsc.edu/).

B. ClinVar (http://www.ncbi.nlm.nih.gov/clinvar), OMIM (http://www.omim.org), SwissVar (http://www.bioinfo.org/wiki/index.php/SwissVar), and the Human Gene Mutation Database (http://www.hgmd.org).

C. SIFT (http://sift.jcvi.org), PROVEAN (http://provean.jcvi.org/index.php), MutationTaster (http://www.mutationtaster.org), PolyPhen-2 (http://genetics.bwh.harvard.edu/pph2), MaxEntScan (http://genes.mit.edu/burgelab/maxent/Xmaxentscan_scoreseq.html), and Human Splicing Finder (http://wwwumd.be/HSF)

D. According to the Z value, children's growth and development were divided into different levels: > + 2 is superior; > + 1 ~ ≤ + 2 is upper middle; + 1 ~ -1 is moderate; ≥ -2 ~ < -1 is below the middle; < -2 is inferior. Assessment tools, such as stadiometers and scales of growth charts, among others, may be obtained from the following websites: http://equipmedical.com.sg/catalog/seca/#all; http://www.progress.com.sg/product/seca-217-stadiometer/; http://www.stadiometer.com/; http://www.healthprofessionalsolutions.com.au. http://www.detecto.com/product/product-category/stadiometers/; http://amamedicalproducts.com.au.
