# Supplementary material for: Chromosome Microarray Analysis and Exome Sequencing: Implementation in Prenatal Diagnosis of Fetuses with Digestive System Malformations
Source: Genes (Basel). 2023 Sep 26;14(10):1872. doi: 10.3390/genes14101872 (PMC10606699; doi:10.3390/genes14101872)
Supplement: Supplementary file 1 [file genes-14-01872-s001.zip › Table S1.pdf]

Table S1 Clinical data of the study cohort.

**Age of pregnant women (median)**	**30.04 years (17.67 - 44.07)**
**Gestational weeks (median)**	**25.14 weeks (13^{+3} -36^{+1})**
**Sex of fetuses (M/F)**	**348/247**
**DSM types**	
Abnormal duodenum morphology	120 (20.2%)
Ascites	109 (18.3%)
Abnormal stomach bubble	96 (16.1%)
Absent gallbladder	42 (7.1%)
Hepatomegaly	33 (5.5%)
Intestinal obstruction	22 (3.7%)
Intestinal duplication	21 (3.5%)
Esophageal atresia	21 (3.5%)
Hepatic hemangioma	15 (2.5%)
Intrahepatic calcification foci	9 (1.5%)
Others*	107 (18.0%)
**Associated other anomalies**	**103 (17.3%)**
**Amniotic fluid anomalies**	**47 (45.6%)**
Polyhydramnios	38
Oligohydramnios	9
**Cardiovascular system**	**25 (24.2%)**
Ventricular septal defect	15
Atrioventricular septal defect	5
Increased cardiothoracic ratio	2
Pericardial effusion	2
Aortic stenosis	1
**Renal anomalies**	**22 (21.4%)**
Enlarged kidneys	10
Multicystic dysplastic kidney	7
Renal cysts	2
Duplex kidney	1
Hydronephrosis	1
Pyelectasis	1
**Skeletal system**	**2 (1.9%)**

Butterfly vertebra	2
Polydactyly	1
**Central nervous system**	**2 (1.9%)**
Ventriculomegaly	1
Arachnoid cyst	1
**Thoracoabdominal wall defects**	**2 (1.9%)**
Omphalocele	1
Diaphragmatic hernia	1
**Others**	**3 (2.9%)**
Fetal growth restriction	2
Hydrops fetalis	1

DSM: digestive system malformations.

*: Others included Abnormality of the intrahepatic bile duct,Meconium peritonitis,Ascites, Intestinal malrotation, Aganglionic megacolon, Splenomegaly,Abdominal mass.
