# Supplementary material for: Chromosome Microarray Analysis and Exome Sequencing: Implementation in Prenatal Diagnosis of Fetuses with Digestive System Malformations
Source: Genes (Basel). 2023 Sep 26;14(10):1872. doi: 10.3390/genes14101872 (PMC10606699; doi:10.3390/genes14101872)
Supplement: Supplementary file 1 [file genes-14-01872-s001.zip › Table S2.pdf]

Table S2 Clinical characteristics of fetuses with digestive system malformations with VUS.

Case	MA (years)	GA (weeks)	Ultrasound findings	Gene	Nucleotide change (amino acid)	Origin	Variant classification	Disorders (OMIM IDs)	Inheritance	Outcomes
1	31.0	30.1	Gastric duplication, Polyhydramnios, Congenital diaphragmatic hernia, Congenital pulmonary airway malformation	ATP7A	NM_000052.7: c.4447G>T (p. Asp1483Tyr) (hemi)	Mother (het)	VUS (PM1+PM2)	Spinal muscular atrophy, distal, X-linked 3(300489), Occipital horn syndrome (304150), Menkes disease (309400)	XLR	TOP
2	29.0	22.7	Ascites, Hepatomegaly, Increased nuchal translucency	TSC2	NM_000548.5: c.2656G>A (p. Val886Met) (het)	Father (het)	VUS (PM2+PP3)	Tuberous sclerosis-2 (613254)	AD	Live birth
3	36.0	21.1	Hepatomegaly, Omphalocele, Increased nuchal translucency, Ventricular septal defect	FLNA	NM_001110556.2: c.5713C>T (p. Pro1905Ser) (hemi)	Mother (het)	VUS (PM1+PM2+PP3+BP1)	Cardiac valvular dysplasia, X-linked (314400), Congenital short bowel syndrome (300048)	XLR/XLD	Live birth
4	29.2	18.0	Biliary atresia, Abnormality of the intrahepatic bile duct	SCN5A	NM_000335.5: c.3944G>A (p. Arg1315Gln) (het)	Mother (het)	VUS (PM1+PM2+PP3)	Heart block, progressive, type IA (11390), Cardiomyopathy, dilated, 1E (601154)	AD	Live birth

5	35.2	26.4	Intestinal malrotation, Volvulus, Intestinal obstruction, FGR	MED12	NM_005120.3: c.2300C>T (p. Ala767Val) (het)	Mother (het)	VUS (PM1+PM2+PP3+BP1)	Ohdo syndrome, X-linked (300895), Lujan-Fryns syndrome (309520), Hardikar syndrome (301068)	XLR/XLD	Live birth
6	29.5	34.1	Abnormal abdomen morphology, Increased nuchal translucency, Ovarian cyst	LRP4	NM_002334.4: c.4505A>C (p. Asp1502Ala) (het)	Father (het)	VUS (PM2+PP3)	Myasthenic syndrome, congenital, 17 (616304),	AR	Live birth
					NM_002334.4: c.1309+5G>C (het)	Mother (het)				
7	30.1	23.6	Ascites, Polyhydramnios, Hydrops fetalis, Talipes equinovarus, Abnormal lung morphology	L1CAM	NM_001278116.2: c.1255A>G (p. Ile419Val) (hemi)	Mother (het)	VUS (PM1+PM2+PP3)	Hydrocephalus, congenital, X-linked (307000), Corpus callosum, partial agenesis of (304100)	XLR/XLD	Live birth
8	29.2	35.0	Tracheoesophageal fistula, Abnormal esophagus morphology, Pleural effusion, Polyhydramnios	MYH11	c.3766A>C (p. Lys1256Gln) (het)	Mother (het)	VUS (PM1+PM2+PP3+BP1)	Aortic aneurysm, familial thoracic 4 (132900), Megacystis-microcolon-intestinal hypoperistalsis syndrome 2 (619351)	AD/AR	Neonatal death
					NM_002474.3: c.3562C>T (p. Arg1188Trp) (het)	Father (het)				
9	38.4	28.1	Hepatic hemangioma, Renal cyst	NPHP4	NM_015102.5: c.3160C>T (p. Arg1054Cy) (het)	Father (het)	VUS (PM1+PP3)	Nephronophthisis 4 (606966), Senior-Loken syndrome 4 (606996)	AR	TOP
					NM_015102.5: c.679G>A (p. Ala227Thr) (het)	Mother (het)				

10	30.3	22.2	Ascites, Pleural effusion, Cardiomyopathy, Cardiomegaly	MYH7	NM_000257.4: c.3658_3660del (p. Glu1220del) (het)	Father (het)	VUS (PS2+PP3+BS2)	Cardiomyopathy, dilated, 1S (613426) Laing distal myopathy (160500)	AD	Live birth
11	32.0	21.7	Cyst of the ductus choledochus, Abnormality of the intrahepatic bile duct, Abnormal biliary tract morphology	SMAD6	NM_005585.5: c.387_388del (p. Ser130GlyfsTer172) (het)	De novo	VUS (PM2+BP4)	Aortic valve disease 2 (614823), Craniosynostosis 7 (617439)	AD	Neonatal death
12	28.1	23.9	Inguinal hernia, Ascites, Hepatomegaly, Hypospadias, Short long bone	SLC25A24	NM_013386.5: c.970T>C (p. Tyr324His) (het)	Father (het)	VUS (PM1+PP3)	Fontaine progeroid syndrome (612289)	AD	Live birth
13	20.3	33.1	Cholestasis, Elevated hepatic transaminase, Cardiomegaly, Coarctation of aorta	TAB2	NM_001292034.3: c.1195C>T (p. Gln399Ter) (het)	Father (het)	VUS (PM1+PP3+BS2)	Congenital heart defects, nonsyndromic, 2 (614980)	AD	Live birth
14	26.4	27.7	Fetal ascites, Echogenic fetal bowel, Cerebellar vermis hypoplasia, Enlarged posterior fossa	MAB21L1	NM_005584.5: c.866C>T (p. Ser289Leu) (het)	Father (het)	VUS (PM2+BP4)	Cerebellar, ocular, craniofacial, and genital syndrome (619479)	AR	TOP
					NM_005584.5: c.576C>T (p. Ile192=) (het)	Mother (het)				
15	36.7	24.7	Abnormal duodenum morphology, Intestinal obstruction	FLNA	NM_001110556.2: c.7000C>T (p. Arg2334Cys) (hemi)	Mother (het)	VUS (PM1+PP3+BS2)	Cardiac valvular dysplasia, X-linked (314400),	XLR/XLD	TOP

								Congenital short bowel syndrome (300048)		
16	31.2	25.2	Abnormal portal venous system morphology, Fetal ascites, Abnormal portal venous system morphology	ABCC6	NM_001171.6: c.4105G>A (p. Glu1369Lys) (het)	Father (het)	VUS (PM2+BP4)	Pseudoxanthoma elasticum (264800), Pseudoxanthoma elasticum, forme fruste (177850)	AD/AR	Live birth
					NM_001171.6: c.557delT (p. Leu186ArgfsTer46) (het)	Mother (het)				
17	29.5	25.0	Polyhydramnios, Esophageal atresia, Preaxial polydactyly, Rib fusion	VPS13B	NM_152564.5: c.365C>T (p. Pro122Leu) (het)	Father (het)	VUS (PM1+PP3+BS2)	Cohen syndrome (216550)	AR	Live birth
					NM_152564.5: c.3865A>G (p. Thr1289Ala) (het)	Mother (het)				
18	34.1	29.9	Polyhydramnios, Fetal ascites, Intrauterine growth retardation	SAMD9	NM_017654.4: c.1195A>G (p. Thr399Ala) (het)	De novo	VUS (PS2+BS2)	MIRAGE syndrome (617053), Tumoral calcinosis, familial, normophosphatemic (610455)	AD/AR	TOP
19	36.5	24.1	Polyhydramnios, Abnormality of the larynx, Abnormality of the hand, Intrauterine growth retardation	AVPR2	NM_000054.4: c.445C>T (p. Arg149Cys) (hemi)	Mother (het)	VUS (PP3+BS2)	Diabetes insipidus, nephrogenic, 1 (304800), Nephrogenic syndrome of inappropriate antidiuresis (300539)	XLR	Live birth
20	37.9	25.9	Intestinal obstruction, Talipes equinovarus, Increased nuchal translucency	AMMECR1	NM_015365.2: c.214C>G (p. Pro72Ala) (hemi)	Mother (het)	VUS (PP3+BS2)	Midface hypoplasia, hearing impairment, elliptocytosis, and nephrocalcinosis (300990)	XLR	Live birth

21	29.2	12.1	Omphalocele, Increased nuchal translucency, Short long bone	NEK1	NM_001199397.3: c.1992delA (p. Val665Cysfs Ter34) (homo)	Father +Mother (het)	VUS (PM1+PP3+BS2)	Short-rib thoracic dysplasia 6 with or without polydactyly (263520), Amyotrophic lateral sclerosis, susceptibility to, 24 (617892)	AD/AR	TOP
22	31.0	32.4	Fetal megacystis, Abnormality of the gallbladder	ACTG2	NM_001615.3: c.86G>A (p. Arg29Gln) (het)	Father (het)	VUS (PM2+BP4)	Visceral myopathy 1 (155310)	AD	Live birth
23	26.3	13.7	Ascites, Fetal cystic hygroma, Pleural effusion, Hypertelorism	LZTR1	NM_006767.3: c.741 C>A (p. Ser247Arg) (het)	Father (het)	VUS (PP3+BS2)	Noonan syndrome 10 (6164564), Schwannomatosis-2 (615670)	AD	Live birth
24	29.6	16.9	Omphalocele, Abnormality of the hand, Abnormality of the foot, Short long bone, Narrow chest	FLNA	NM_001110556: c.583 G>A (p. G195S) (hemi)	Mother (het)	VUS (PP3+BS2)	Cardiac valvular dysplasia, X-linked (314400), Congenital short bowel syndrome (300048)	XLR/XLD	Live birt
25	34.1	25.7	Anal atresia, Esophageal atresia, Polyhydramnios, Ventricular septal defect	DACT1	NM_016651: c.74C>T (p.A25V) (het)	De novo	VUS (PM1+PP3+BS2)	Townes-Brocks syndrome 2 (617466)	AD	TOP
26	24.0	34.1	Splenomegaly, Cardiomegaly	ADCY5	NM_183357: c.3388A>T (p. M1130L) (het)	Mother (het)	VUS (PM2+BP4)	Dyskinesia with orofacial involvement, autosomal dominant (606703)	AD	Live birth

hemi: hemizygous; het: heterozygous; homo: homozygous; VUS: variants of unknown significance; MA: maternal age; GA: gestational age; TOP: termination of pregnancy; AD: autosomal dominant; AR: autosomal recessive; XLD: X-linked dominant; XLR: X-linked recessive;
